# Pharmacokinetic Interactions between Primaquine and Chloroquine

**DOI:** 10.1128/AAC.02794-13

**Published:** 2014-06

**Authors:** Sasithon Pukrittayakamee, Joel Tarning, Podjanee Jittamala, Prakaykaew Charunwatthana, Saranath Lawpoolsri, Sue J. Lee, Warunee Hanpithakpong, Borimas Hanboonkunupakarn, Nicholas P. J. Day, Elizabeth A. Ashley, Nicholas J. White

**Affiliations:** a Faculty of Tropical Medicine, Mahidol University, Bangkok, Thailand; b Mahidol Oxford Tropical Medicine Research Unit, Faculty of Tropical Medicine, Mahidol University, Bangkok, Thailand; c Centre for Tropical Medicine, Nuffield Department of Medicine, University of Oxford, Oxford, United Kingdom

## Abstract

Chloroquine combined with primaquine has been the standard radical curative regimen for Plasmodium vivax and Plasmodium ovale malaria for over half a century. In an open-label crossover pharmacokinetic study, 16 healthy volunteers (4 males and 12 females) aged 20 to 47 years were randomized into two groups of three sequential hospital admissions to receive a single oral dose of 30 mg (base) primaquine, 600 mg (base) chloroquine, and the two drugs together. The coadministration of the two drugs did not affect chloroquine or desethylchloroquine pharmacokinetics but increased plasma primaquine concentrations significantly (*P* ≤ 0.005); the geometric mean (90% confidence interval [CI]) increases were 63% (47 to 81%) in maximum concentration and 24% (13 to 35%) in total exposure. There were also corresponding increases in plasma carboxyprimaquine concentrations (*P* ≤ 0.020). There were no significant electrocardiographic changes following primaquine administration, but there was slight corrected QT (QTc) (Fridericia) interval lengthening following chloroquine administration (median [range] = 6.32 [−1.45 to 12.3] ms; *P* < 0.001), which was not affected by the addition of primaquine (5.58 [1.74 to 11.4] ms; *P* = 0.642). This pharmacokinetic interaction may explain previous observations of synergy in preventing P. vivax relapse. This trial was registered at ClinicalTrials.gov under reference number NCT01218932.

## INTRODUCTION

Chloroquine, together with primaquine, has been the standard regimen for the radical cure of Plasmodium vivax and Plasmodium ovale malaria since the early 1950s (1, 2). Chloroquine is a highly effective schizontocide, while primaquine has weaker asexual-stage activity (3) but is the only generally available drug with hypnozoitocidal properties, i.e., it kills dormant liver stage parasites and prevents relapse (radical cure) (1). Despite their extensive use as coadministered drugs in many millions of people, pharmacokinetic interactions between the two drugs have never been investigated. Important pharmacokinetic interactions were documented between their predecessors, mepacrine (also known as quinacrine or atebrine), which is structurally related to chloroquine, and pamaquine (plasmoquine), which is structurally related to primaquine (4, 5). The result was substantial elevations in serum pamaquine concentrations, which were associated with increased toxicity. This suggests that the elevated concentrations were also associated with increased production of the reactive metabolites thought to mediate the biological effects of the 8-aminoquinolines. Alving and colleagues provided evidence of synergy in radical curative activity between primaquine and both quinine and chloroquine (6). Primaquine is metabolized mainly to carboxyprimaquine via monoamine oxidase and via cytochrome P450 enzymes to several other identified and unidentified metabolites that are detectable in plasma or urine (7). The cytochrome P450 2D6 route of metabolism is believed to generate the reactive intermediates that mediate the hypnozoitocidal effect (7). The same reactive metabolites are believed to be responsible for oxidant hemolysis in patients with glucose-6-phosphate dehydrogenase (G6PD) deficiency. The present study was conducted in healthy volunteers to investigate the potential pharmacokinetic interactions between primaquine and chloroquine.

## MATERIALS AND METHODS

### Participants.

Sixteen healthy Thai adult volunteers were studied in an open-label, two-arm crossover, three-period, randomized pharmacokinetic comparison conducted in the volunteer facility of the Hospital for Tropical Diseases, Faculty of Tropical Medicine, Mahidol University, Bangkok, Thailand. The inclusion criteria were nonpregnant adults with normal G6PD activity aged 20 to 60 years, body mass indices of 18 to 25 kg of body weight/m^2^, and a normal electrocardiogram with corrected QT (QTc) (using Fridericia's correction) of <450 ms. All volunteers were HIV-negative, non-hepatitis B- and C-infected nonsmokers; did not take alcohol to excess; and had not taken chloroquine or primaquine for at least 12 months before the study. All participants clearly understood the purpose of the study and provided written informed consent.

The study was approved by the Ethics Review Committee of the Faculty of Tropical Medicine, Mahidol University (TMEC 10-017), and by the Oxford University Tropical Research Ethics Committee (OxTREC 39-10). The trial was registered at ClinicalTrials.gov under reference number NCT01218932.

### Sample size calculation.

The sample size was determined in order to evaluate the potential pharmacokinetic drug-drug interaction between primaquine and chloroquine, reflected primarily by the areas under the plasma concentration-time curve (AUCs). Absence of a significant interaction was considered to have been demonstrated if the 90% confidence interval (CI) for the geometric mean ratios of AUC_0-last_ (total drug exposure up to the last observation) and AUC_0-∞_ (total drug exposure extrapolated to infinity) for the combination versus the single drug were within the range of 0.80 to 1.25 (8, 9). Based on earlier pharmacokinetic studies (10–17), it was estimated that enrolling 16 subjects, to ensure a sample size of 13 subjects completing the full study, would provide at least 80% power to detect with 95% confidence a 30% increase or decrease in the primaquine AUC_0-∞_.

### Drug administration.

This was a three-way, two-arm crossover study of the oral regimens. All participants were allocated, using a computer-generated randomization list, to receive 30 mg base primaquine (primaquine phosphate in two tablets with 26.34 mg salt/tablet, equivalent to 15 mg base/tablet; Thailand Government Pharmaceutical Organization) to be followed at the second visit by either 600 mg base chloroquine (chloroquine phosphate in four tablets; 150 mg base/tablet; Thailand Government Pharmaceutical Organization) or chloroquine plus primaquine in the same doses. The opposite regimen (chloroquine or chloroquine-primaquine) was given to complete the crossover at the third visit. The washout period following primaquine alone was at least 7 days, and following chloroquine or chloroquine-primaquine it was 8 weeks. All regimens were given under supervision 30 min after a light meal following an overnight fast. Food was then restricted for 4 h. Any subject who vomited within 10 min of administration of the study drugs was to be withdrawn.

### Study assessments and blood sampling.

The volunteers were admitted to the volunteer facility at the Hospital for Tropical Diseases, Bangkok, Thailand, on the evening before each study period began. On enrollment and each morning, the subjects were asked about their state of health and well-being, adherence to study restrictions, and use of any concomitant medication since screening or the previous study visit. Adverse events, hematology, clinical chemistry, urinalysis, electrocardiograms (ECGs), and vital-sign results were recorded on a standard case record form throughout each treatment period. The subjects were discharged after completion of the study procedures at 24 h. Vital signs and electrocardiograms were recorded at 0, 1, 2, 4, 8, 12, and 24 h after drug administration. Methemoglobinemia was monitored at each blood-sampling time using a noninvasive methemoglobin monitor associated with a Masimo pulse oximeter, SpMet. Blood samples (2 ml) were drawn into fluoride oxalate tubes via individual venipunctures or an indwelling intravenous cannula during the intensive sampling phase (up to 24 h after dosing). Samples for plasma drug measurement were collected at predose and then at 0.25, 0.5, 1, 1.5, 2, 3, 4, 6, 8, 10, 12, and 24 h postdose. Additional blood samples were taken for plasma chloroquine measurements on days 3, 4, 7, 11, 15, 22, and 36. After collection, the blood samples were centrifuged for 10 min at 2,000 × *g* at 4°C, and the plasma was transferred to a prelabeled sample storage tube, stored upright in a non-self-defrosting freezer at −70°C or lower, and transferred immediately to the Department of Clinical Pharmacology, Mahidol Oxford Tropical Medicine Research Unit, Bangkok, Thailand, for drug measurements. The laboratory participates in the WorldWide Antimalarial Resistance Network (WWARN) quality control and assurance proficiency-testing program with satisfactory performance (http://www.wwarn.org/toolkit/qaqc).

### Drug quantification.

Chloroquine and desethylchloroquine plasma concentrations were quantified using solid-phase extraction and high-performance liquid chromatography with UV detection (18). The limit of quantification was 5 ng/ml for both.

Primaquine and carboxyprimaquine plasma concentrations were quantified using a validated assay according to U.S. FDA guidelines (unpublished data). Briefly, samples were preprocessed by protein precipitation, followed by phospholipid removal solid-phase extraction. Analytes were separated using reversed-phase high-performance liquid chromatography and quantified by electrospray ionization in the positive mode with multireaction monitoring mass spectrometry detection. The method did not show any evidence of significant ion suppression/enhancement for primaquine or carboxyprimaquine. Stable isotope-labeled internal standards were used to compensate for any unexpected matrix effects. The absolute recovery was approximately 70% at all tested concentrations for primaquine, carboxyprimaquine, and their internal standards. Within-day and between-day precision for primaquine and carboxyprimaquine were below 9% at all tested quality control concentrations and below 15% at the lower limit of quantification. The limits of quantification were 1.14 ng/ml and 4.88 ng/ml for primaquine and carboxyprimaquine, respectively.

Three replicates of quality control samples at low, middle, and high concentrations were analyzed within each batch of clinical samples to ensure precision and accuracy during drug measurements. Total precision (i.e., relative standard deviation [SD]) for all drug measurements was below 7% during drug quantification.

### Safety analysis.

Safety was assessed based on adverse events, physical examination, vital signs, clinical laboratory parameters, electrocardiographic changes, and methemoglobin levels. A 30-ms or more change from baseline in QTc (using Fridericia's correction) was specified prospectively as potentially clinically significant.

The safety and tolerability of primaquine, chloroquine, and the combined regimens were assessed by pooled comparison of all visits by treatment group using the Wilcoxon matched-pairs signed-rank test for continuous variables or McNemar's exact test for categorical variables. The frequencies (percentages) of adverse events and serious adverse events were presented by treatment group and reported by visit. All biochemical variables were compared using the Mann-Whitney U test and within groups (to assess reexposure to chloroquine) using the Wilcoxon matched-pairs signed-rank test.

### Pharmacokinetic analysis.

Individual plasma concentration-time data were evaluated using a noncompartmental analysis approach in WinNonlin version 5.3 (Pharsight Corporation, CA, USA). Total exposure up to the last measured concentration (AUC_0-last_) was calculated using the linear trapezoidal method for ascending concentrations and the logarithmic trapezoidal method for descending concentrations. The terminal elimination half-life (*T*_1/2_) was estimated from the slope (λ_*Z*_) of the best-fit log-linear regression of the observed concentrations in the terminal elimination phase. Drug exposure was extrapolated from the last observed concentration (*C*_last_) to infinity by *C*_last_/λ_*Z*_ for each individual subject to compute total drug exposure (AUC_0-∞_). The maximum plasma concentration (*C*_max_) and time to maximum concentration (*T*_max_) were taken directly from the observed data. Oral clearance (CL/*F*) and the apparent volume of distribution (*V_Z_*/*F*) were computed individually according to the following equations: CL/*F* = dose/AUC and *V_Z_*/*F* = [dose/(ln_2_/*T*_1/2_)] × AUC.

Complete *in vivo* conversion of primaquine into carboxyprimaquine and of chloroquine into desethylchloroquine were assumed for these calculations. The administered doses of carboxyprimaquine and desethylchloroquine were calculated using the relative differences in molecular weights.

Pharmacokinetic parameter estimates were compared between a single dose of each drug administered alone and in combination with the other drug using the paired Wilcoxon signed-rank test in STATA v.11. Analysis of variance (ANOVA) was carried out on the log-transformed pharmacokinetic parameters *C*_max_, AUC_0-last_, and AUC_0-∞_ to assess the bioequivalence of drug administered alone and in combination. Bioequivalence was assumed if the 90% confidence intervals of the geometric mean ratios (combination/alone) of *C*_max_, AUC_0-last_, and AUC_0-∞_ were within 80% to 125% (8, 9).

## RESULTS

### Subjects.

There were 4 males and 12 females aged 20 to 47 years enrolled in the study (demographics and laboratory results are shown in Table 1). For all three visits, all subjects were healthy by history, physical examination, vital signs, electrocardiograms, and laboratory investigations. The two groups (for the sequence of drug administration) were compatible, as there were no significant differences in gender distribution, age, body weight, or baseline laboratory investigations (*P* ≥ 0.123).

**TABLE 1:** Baseline characteristics stratified by treatment group^*a*^

Characteristic	Value^*b*^		
	Group A (*n* = 8)	Group B (*n* = 8)	Total (*n* = 16)
Gender (no. of males/no. of females)	3/5	1/7	4/12
Age (yr)	37.8 ± 7.5	32.8 ± 8.1	35.3 ± 8.0
Wt (kg)	61.0 ± 5.6	59.9 ± 8.0	60.4 ± 6.7
Hematocrit (%)	39.8 (36.3– 46.5)	37.5 (36.5–43.5)	39 (36.3–46.5)
White blood cell count (×10^3^/μl)	6.9 (5.2–8.6)	7.0 (3.8–8.5)	7.0 (3.8–8.6)
Total bilirubin (mg/dl)	0.55 (0.26–0.83)	0.41 (0.15–0.63)	0.44 (0.15–0.83)
Alanine aminotransferase (U/liter)	16 (12–34)	12 (7–27)	14 (7–34)
Aspartate aminotransferase (U/liter)	20 (15–31)	19 (14–27)	19 (14–31)
Serum creatinine (mg/dl)	0.7 (0.5–0.9)	0.7 (0.6–1.0)	0.7 (0.5–1.0)
Methemoglobin (%)	0.8 (0.1–1.6)	1.2 (0.8–1.8)	1.1 (0.1–1.8)
Pulse rate (per min)	70 (55–85)	64 (52–75)	67 (52–75)
QTc interval (ms)	406 (362–437)	398 (377–425)	410 (341–456)

a This was a three-way, two-arm crossover study. The sequence of oral regimens for group A was primaquine, primaquine-chloroquine, and chloroquine, and for Group B it was primaquine, chloroquine, and primaquine-chloroquine.

b Data are reported as mean ± SD or median (range).

### Safety profiles following drug administration.

All 16 subjects completed the three study visits with good adherence to study procedures. None of the subjects experienced nausea or vomited. There were no drug rashes or other serious adverse effects in any of the subjects during or after the study visits. Liver enzyme levels were normal for all subjects throughout the 2-month study period. None had abnormal methemoglobin levels. Overall, there was a small but statistically significant lengthening of the QTc (Fridericia) interval following chloroquine administration (*P* < 0.001) but no change with primaquine (*P* = 0.278). The addition of primaquine to chloroquine had no effect on the QTc prolongation (*P* = 0.642) (Table 2). The longest QT interval recorded in the study was in a woman whose baseline QT interval was 438 ms and rose to 462 ms at 8 h after oral chloroquine administration (heart rate, 66/minute on both occasions).

**TABLE 2:** Maximum changes from baseline in electrocardiograph QTc (Fridericia) intervals within 24 h after drug administration

Parameter	Value^*a*^			*P* value		
	PQ alone (*n* = 16)	CQ alone (*n* = 16)	PQ-CQ combination (*n* = 16)	PQ vs. CQ	PQ vs. PQ-CQ	CQ vs. PQ-CQ
Onset (h)	8 (1–24)	8 (2–24)	8 (1–12)	0.7356	0.3979	1.0
Median QTc change from baseline (ms)	1.42 (−2.85–5.31)	6.32 (−1.45–12.3)	5.58 (1.74–11.4)	0.0032	0.0013	0.6417
Mean QTc change from baseline (ms)	1.20 ± 2.33	6.10 ± 3.67	6.14 ± 2.48	0.0010	0.0001	0.9699

a Data are presented as median (range) or mean ± SD. PQ, primaquine; CQ, chloroquine.

### Primaquine/carboxyprimaquine pharmacokinetics.

**FIG 1:**
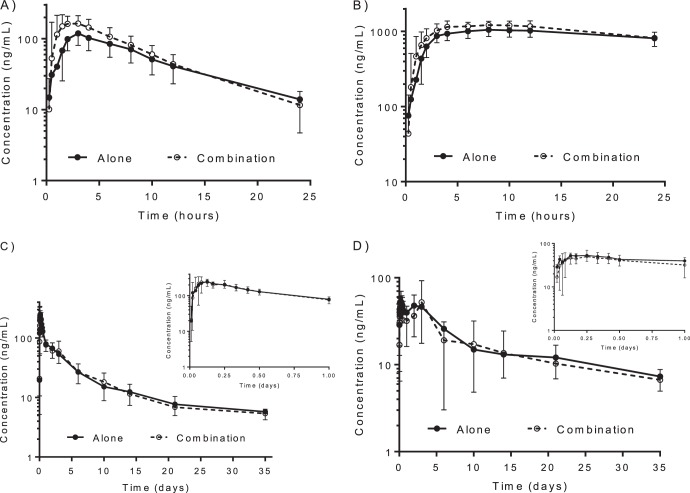
Mean (±SD) pharmacokinetic profiles of primaquine (A), carboxyprimaquine (B), chloroquine (C), and desethylchloroquine (D) after a single oral dose alone or in combination. The insets illustrate concentration-time profiles during the first day of dosing.

**FIG 2:**
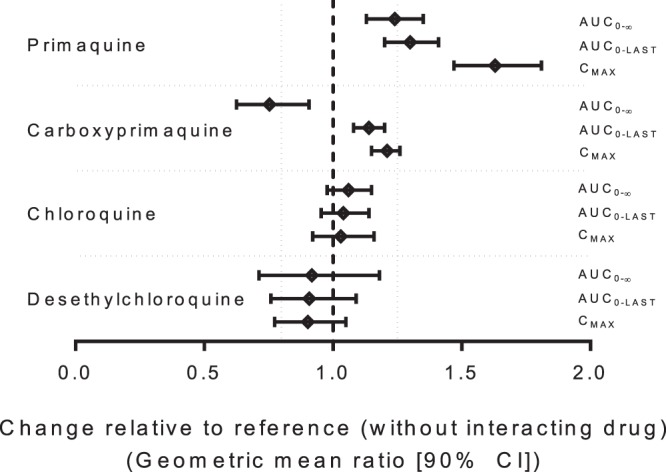
Forest plot illustrating the degree of drug-drug interaction after administration of primaquine and chloroquine as a single oral dose alone or in combination. The carboxyprimaquine AUC_0-∞_ estimate was biased by three outliers in which over 90% of the AUC was extrapolated.

Primaquine and carboxyprimaquine pharmacokinetics were well described using a noncompartmental analysis (Table 3 and Fig. 1). Administration of primaquine in combination with chloroquine resulted in significantly lower primaquine CL/*F* (*P* = 0.0005) and *V*/*F* (*P* = 0.0004) values than with primaquine administered alone, leading to significantly higher drug exposure (*C*_max_, *P* = 0.0004; AUC_0-last_, *P* = 0.0004; and AUC_0-∞_, *P* = 0.0005). Coadministration of primaquine together with chloroquine also shortened the primaquine *T*_1/2_ (*P* = 0.0004) (Table 3). The geometric means (90% confidence intervals) of the combination/alone ratios for the logarithmically transformed values of primaquine *C*_max_, AUC_0-last_, and AUC_0-∞_ were 163% (147% to 181%), 130% (120% to 141%), and 124% (113% to 135%), respectively (Table 4 and Fig. 2). Thus, the geometric mean ratios and 90% confidence intervals of the ratios were outside the 80% to 125% range for assuming bioequivalence of primaquine.

**TABLE 3:** Noncompartmental pharmacokinetic analysis of primaquine and carboxyprimaquine when primaquine was administered alone or in combination with chloroquine

Parameter^*a*^	Value^*b*^					
	Primaquine			Carboxyprimaquine		
	Alone (*n* = 16)	Combination (*n* = 16)	*P* value	Alone (*n* = 16)	Combination (*n* = 16)	*P* value
Total dose (mg/kg)	0.485 (0.426–0.641)	0.485 (0.426–0.641)	NA	0.513 (0.450–0.678)	0.513 (0.450–0.678)	NA
*T*_lag_ (h)	0.25 (0– 0.5)	0.25 (0–0.5)	0.4010	0.25 (0–0.50)	0 (0–0.50)	0.0480
*C*_max_ (ng/ml)	122 (50.1–215)	208 (109–424)	0.0004	1,080 (650–1,420)	1,300 (987–1,660)	0.0004
*T*_max_ (h)	3.00 (1.00–3.00)	2.00 (0.50–4.00)	0.0238	8.00 (4.00–12.0)	8.00 (4.00–12.0)	0.7312
CL/*F* (liters/h)	25.3 (13.0–53.0)	20.9 (11.3–31.5)	0.0005	0.560 (0.102–0.862)	0.549 (0.328–0.899)	0.0200
*V*/*F* (liters)	247 (154–544)	162 (96.5–271)	0.0004	25.6 (19.6–43.6)	21.5 (14.6–27.6)	0.0004
*T*_1/2_ (h)	6.63 (5.10–10.3)	5.90 (4.11–7.80)	0.0004	33.2 (20.4–165)	24.8 (12.9–45.9)	0.0011
AUC_0-last_ (h · ng/ml)	1,100 (500–1,970)	1,360 (883–2,420)	0.0004	21,300 (13,100–26,800)	24,300 (18,400–32,800)	0.0011
AUC_0-∞_ (h · ng/ml)	1,190 (566–2,310)	1,440 (953–2,650)	0.0005	56,700 (36,800–312,000)	57,900 (35,300–96,800)	0.0229
Ext. AUC (%)	9.04 (4.21–22.1)	5.65 (1.91–12.2)	0.0004	64.2 (47.8–91.7)	54.7 (36.0–72.2)	0.0009

a *T*_lag_, observed lag time to absorption; *C*_max_, maximum observed plasma concentration after oral administration; *T*_max_, observed time to reach *C*_max_; CL, elimination clearance; *V*, apparent volume of distribution; *T*_1/2_, terminal elimination half-life; AUC_0-last_, observed area under the plasma concentration-time curve from zero time to last observed concentration; AUC_0-∞_, predicted area under the plasma concentration-time curve after the last dose from zero time to infinity; Ext. AUC, percentage of AUC_0-∞_ extrapolated from the last observation to infinity; and *F*, oral bioavailability.

b Data are presented as median (range) with *P* values calculated using the paired Wilcoxon signed-rank test. NA, not available.

**TABLE 4:** Noncompartmental pharmacokinetic analysis of chloroquine and desethylchloroquine when chloroquine was administered alone or in combination with primaquine

Parameter	Value^*a*^					
	Chloroquine			Desethylchloroquine		
	Alone (*n* = 16)	Combination (*n* = 16)	*P* value	Alone (*n* = 16)	Combination (*n* = 16)	*P* value
Total dose (mg/kg)	9.69 (8.51–12.8)	9.69 (8.51–12.8)	NA	8.84 (7.76–11.7)	8.84 (7.76–11.7)	NA
*T*_lag_ (h)	0.25 (0–0.50)	0.25 (0–0.50)	0.3651	0.75 (0.25–1.50)	0.50 (0.25–1.50)	0.2309
*C*_max_ (ng/ml)	295 (110–496)	290 (166–650)	0.7960	73.7 (33.6–121)	56.4 (36.9–152)	0.1961
*T*_max_ (h)	3.00 (1.00–6.00)	2.00 (1.00–6.00)	0.4199	35.2 (1.00–143)	9.00 (1.5–71.0)	0.5014
CL/*F* (liters/h)	40.0 (24.5–56.5)	37.6 (21.4–52.9)	0.2146	34.6 (18.3–87.6)	43.2 (15.8–123)	0.1337
*V*/*F* (liters)	7,600 (4,450–12,400)	9,170 (5,850–33,400)	0.0437	13,000 (7,540–61,000)	17,700 (6,370–54,100)	0.7564
*T*_1/2_ (h)	134 (82.9–329)	168 (85.4–615)	0.0174	312 (88.7–1,200)	295 (86.6–2,380)	0.7174
AUC_0-last_ (h · μg/ml)	14.0 (9.50–20.8)	14.0 (10.7–25.9)	0.5349	11.6 (5.45–22.7)	10.1 (3.62–28.3)	0.4691
AUC_0-∞_ (h · μg/ml)	15.0 (10.6–24.5)	16.0 (11.3–28.0)	0.2553	15.8 (6.25–29.9)	12.7 (4.47–34.7)	0.1477
Ext. AUC (%)	7.15 (1.69–15.2)	7.67 (2.83–24.2)	0.3011	16.9 (8.70–55.1)	17.4 (4.79–74.2)	0.7564
Day 7 plasma concn (ng/ml)	28.1 (12.8–50.1)	23.1 (14.4–51.4)	0.8361	19.4 (5.76–91.3)	16.6 (6.73–49.6)	0.3794
Day 14 concn (ng/ml)	11.4 (5.80–26.0)	10.2 (5.36–26.1)	0.9176	11.9 (4.84–22.5)	11.5 (4.01–39.1)	0.6791

a Data are presented as median (range) with *P* values calculated using the paired Wilcoxon signed-rank test.

Primaquine is metabolized rapidly in the liver to carboxyprimaquine. Administration of primaquine in combination with chloroquine resulted in significantly higher carboxyprimaquine exposures (*C*_max_, *P* = 0.0004; AUC_0-last_, *P* = 0.0011; and AUC_0-∞_, *P* = 0.0229), lower CL/*F* (*P* = 0.0200) and *V*/*F* (*P* = 0.0004) values, and shorter *T*_1/2_ (*P* = 0.0011) than when administered alone (Table 3). The accuracy of the carboxyprimaquine half-life estimates was limited, and thus, the comparison of AUC_0-∞_ estimates was unreliable, as most of the carboxyprimaquine AUCs were extrapolated (Fig. 1), and the geometric mean value was biased by three outlying estimates in which over 90% of the AUC was extrapolated (Fig. 2). The geometric mean percentages (90% confidence intervals) of the combination/alone ratio for the logarithmically transformed values of carboxyprimaquine *C*_max_, AUC_0-last_, and AUC_0-∞_ were 121% (115% to 126%), 114% (108% to 120%), and 75.3% (62.5% to 90.7%), respectively (Table 4 and Fig. 2). Thus, the geometric mean ratios and 90% confidence intervals of the ratios of *C*_max_ and AUC_0-∞_ were also outside the 80% to 125% range for assuming bioequivalence of carboxyprimaquine.

### Chloroquine/desethylchloroquine pharmacokinetics.

Chloroquine and desethylchloroquine pharmacokinetics were well described using a noncompartmental analysis (Table 4 and Fig. 1). Administration of chloroquine in combination with primaquine resulted in significantly higher chloroquine *V*/*F* values (*P* = 0.0437) and longer *T*_1/2_ (*P* = 0.0174) but with higher interindividual variation than with chloroquine administered alone (Table 4). However, the geometric mean percentages (90% confidence intervals) of the combination/alone ratio for the logarithmically transformed values of chloroquine *C*_max_, AUC_0-last_, and AUC_0-∞_ were 103% (92.1% to 116%), 104% (95.4% to 114%), and 106% (97.7% to 115%), respectively (Table 4 and Fig. 2). Thus, the geometric mean ratios and 90% confidence intervals of the ratios met the U.S. FDA criteria for assuming bioequivalence of chloroquine. There was also no significant difference in desethylchloroquine pharmacokinetics when chloroquine was administered alone or in combination with primaquine. The geometric mean percentages (90% confidence intervals) of the combination/alone ratios for the logarithmically transformed values of desethylchloroquine *C*_max_, AUC_0-last_, and AUC_0-∞_ were 90.2% (77.3% to 105%), 90.8% (75.9% to 109%), and 91.8% (71.2% to 118%), respectively (Table 5 and Fig. 2). However, there was too much variability to assume bioequivalence of desethylchloroquine.

**TABLE 5:** Bioequivalence analysis of primaquine, carboxyprimaquine, chloroquine, and desethylchloroquine after primaquine and chloroquine administered as a single oral dose alone and in combination

Parameter	Value^*a*^			
	Primaquine (*n* = 16)	Carboxyprimaquine (*n* = 16)	Chloroquine (*n* = 16)	Desethylchloroquine (*n* = 16)
*C*_max_ (ng/ml)	163 (147–181)	121 (115–126)	103 (92.1–116)	90.2 (77.3–105)
AUC_0-last_ (h · ng/ml)	130 (120–141)	114 (108–120)	104 (95.4–114)	90.8 (75.9–109)
AUC_0-∞_ (h · ng/ml)	124 (113–135)	75.3 (62.5–90.7)	106 (97.7–115)	91.8 (71.2–118)

a Data are presented as geometric mean ratios (90% CI).

## DISCUSSION

Chloroquine and primaquine have been used together for radical cure since the early 1950s and thus represent the longest-serving currently deployed antimalarial treatment. Chloroquine has potent asexual-stage activity and is still a first-line treatment for P. vivax, P. malariae, P. ovale, and P. knowlesi malaria, although resistance is increasing in P. vivax. Primaquine has weaker asexual-stage activity (2, 19) but potent preerythrocytic and radical curative activities. Together, they provide a combination therapy for blood stage P. vivax and *P. ovale* infections, and primaquine eliminates the liver stage parasites. The long courses of primaquine required for radical cure of P. vivax and P. ovale malaria have seldom been used without chloroquine. Primaquine is effectively a prodrug generating reactive intermediate oxidative metabolites via cytochrome P450 biotransformation, which mediate biological activity (antiparasitic effects and hemolytic toxicity) (7, 19). The metabolic pathway via monoamine oxidase to carboxyprimaquine is not thought to be directly relevant to biological activity, as the metabolite is inert (7).

This crossover study in healthy volunteers gave pharmacokinetic estimates for primaquine that are generally similar to those reported previously (11–16). The interaction evaluation shows that chloroquine has significant effects on primaquine pharmacokinetics, whereas primaquine has negligible effects on the disposition of chloroquine. Coadministration of chloroquine increased peak primaquine concentrations by approximately 63% and overall primaquine exposure by 24%. Carboxyprimaquine levels were also correspondingly elevated, although the sampling design did not allow an accurate estimation of total exposures. This profile suggests displacement from tissue binding sites by chloroquine with consequent contraction in the apparent volume of distribution. However, a contraction of the apparent volume of distribution cannot explain the increased total exposure of primaquine and suggests additional mechanistic interactions affecting primaquine absorption or elimination clearance. Primaquine has been reported to be almost completely absorbed (96%) after oral administration (11), so a drug-drug interaction resulting in increased absorption could explain only a very small fraction of the increased exposure. Primaquine displays a moderately high-affinity binding to alpha-1 acid glycoprotein, whereas chloroquine shows a weak binding affinity to the protein, so concomitant administration is not expected to result in a significant drug-drug interaction due to plasma protein displacement (20). A cytochrome P450-mediated interaction resulting in decreased elimination clearance of primaquine (i.e., higher exposure) when coadministered is possible. The disproportionate decrease in the elimination clearance and apparent volume of distribution resulted in an overall shortening of the terminal elimination half-life when coadministered (Table 3).

Although the disposition of the parent drug and metabolites within the infected hepatocyte cannot be extrapolated directly, the earlier observation of apparent therapeutic synergy noted by Alving and colleagues does suggest that chloroquine potentiates the antirelapse effect of primaquine (6). However, these early pharmacodynamic interaction studies suggested that quinine also potentiated the action of primaquine, but pharmacokinetic interaction studies with quinine using less sensitive assays demonstrated no significant interaction with primaquine, although they did show that quinine elevated carboxyprimaquine levels (17). Nevertheless, taken together with the well-documented toxic interaction between mepacrine (quinacrine) and pamaquine (4, 5) and the complete lack of hypnozoitocidal activity with chloroquine alone, these data suggest that the potentiation of the radical curative activity of primaquine by chloroquine has a pharmacokinetic basis.

The converse effect of primaquine on chloroquine disposition was modest in comparison. Chloroquine caused a slight lengthening of the electrocardiographic QTc interval, as has been reported previously (21), and this was not affected by primaquine. Whether chloroquine potentiates the hemolytic toxicity of primaquine in G6PD deficiency remains to be determined.

If either chloroquine or primaquine was a newly developed drug, these interactions would have been well characterized by the pharmaceutical industry before clinical deployment. This belated discovery, after more than half a century of widespread use in millions of people, that the two most widely used drugs for the treatment of vivax malaria interact with each other testifies to the limited support for clinical pharmacology research in the public sector in tropical countries.
